# A Treatise on Diseases of the Nervous System

**Published:** 1876-07

**Authors:** 


*A Treatise on Diseases of the Nervous System.* By Wm. A. Hammond, M. D. Sixth Edition, re-written, enlarged and improved. New York: D. Appleton & Co., 1876. Buffalo: Martin Taylor.

This work has been almost wholly re-written since the appearance of the last edition, and much additional matter has been incorporated within its pages. In its present condition the work of Dr. Hammond, seems as complete as any with which we are acquainted, the description of the various forms of nervous disorders is, in the main, excellent, and the treatment advised by the author judicious; his readers must not however be disappointed if the results which they obtain are not what the text would lead them to anticipate.

While we can commend the labor of the author in so thoroughly revising and adding to the text of his truly valuable work, we can not say as much in praise of the judgement exercised in the selection of the illustrations for certain portions of the work; those on pages 769–770–771 for instance, have too much of a sensational appearance, and while doubtless true representations are not necessary to simplify the text.

